# PEI-Engineered Respirable Particles Delivering a Decoy Oligonucleotide to NF-κB: Inhibiting MUC2 Expression in LPS-Stimulated Airway Epithelial Cells

**DOI:** 10.1371/journal.pone.0046457

**Published:** 2012-10-03

**Authors:** Francesca Ungaro, Daniela De Stefano, Concetta Giovino, Alessia Masuccio, Agnese Miro, Raffaella Sorrentino, Rosa Carnuccio, Fabiana Quaglia

**Affiliations:** 1 Department of Pharmaceutical and Toxicological Chemistry, University of Naples Federico II, Naples, Italy; 2 Department of Experimental Pharmacology, University of Naples Federico II, Naples, Italy; National Jewish Health, United States of America

## Abstract

A specific and promising approach to limit inflammation and mucin iperproduction in chronic lung diseases relies on specific inhibition of nuclear Factor-κB (NF-κB) by a decoy oligonucleotide (dec-ODN). To fulfill the requirements dictated by translation of dec-ODN therapy in humans, inhalable dry powders were designed on a rational basis to provide drug protection, sustained release and to optimize pharmacological response. To this end, large porous particles (LPP) for dec-ODN delivery made of a sustained release biomaterial (poly(lactic-co-glycolic) acid, PLGA) and an “adjuvant” hydrophilic polymer (polyethylenimine, PEI) were developed and their effects on LPS-stimulated human airway epithelial cells evaluated. The composite PLGA/PEI particles containing dec-ODN (i.e., LPP_PEI_) were successfully engineered for widespread deposition in the lung and prolonged release of intact dec-ODN *in vitro*. LPP_PEI_ caused a prolonged inhibition of IL-8 and MUC2 expression in CF human bronchial epithelial cells and human epithelial pulmonary NCI-H292 cells, respectively, as compared to naked dec-ODN. Nonetheless, as compared to previously developed LPP, the presence of PEI was essential to construct a dec-ODN delivery system able to act in mucoepidermoid lung epithelial cells. In perspective, engineering LPP with PEI may become a key factor for tuning carrier properties, controlling lung inflammation and mucin production which, in turn, can foster *in vivo* translation of dec-ODN therapy.

## Introduction

Recognition of the global importance and the rising prevalence of chronic respiratory diseases, such as cystic fibrosis (CF), and the absence of effective therapeutic treatments, has recently prompted a great deal of research efforts into the development of new oligonucleotide (ON) therapeutics, addressing the underlying pathology of lung diseases [1], [2] Among molecular targets, the nuclear factor-κB (NF-κB) transcriptionally regulates the expression of several inflammatory mediators, such as cytokines and chemokines (e.g., IL-6 and IL-8) [3]. In particular, persistent NF-κB activation has been reported both in the presence and absence of pathogens in the airways of CF patients and *in vitro* [4], [5]. In addition, inflammatory mediators contribute to the overproduction of mucins/mucus, responsible for airway obstruction and mucociliary function failure in CF patients [6]–[9]. Thus, NF-κB blockade by decoy ONs has recently been proposed as a strategy to limit the progression of chronic lung inflammation in CF [10]. Nonetheless, the development of adequate delivery systems remains an important challenge to translate ON-based therapies from bench to bedside.

Taking a look at the ON therapeutic pipelines for chronic lung diseases, it is apparent that the most widely employed dosage forms for ON delivery are solutions for injections or inhalation [1], [2]. Of course, pulmonary application of ONs should be the choice to maintain high local concentrations, which is relevant when the lung is the target organ. Nonetheless, extensive *in vitro*/*in vivo* degradation (particular dramatic in case of double-stranded decoy ONs and siRNA) as well as their difficult translocation in lung lining fluids and poor uptake by target epithelial cells. In fact, the failure of nucleic acid delivery to the airway epithelia is largely attributed to extracellular and cellular barriers [2], [11], [12]. In particular, the thick and tenacious CF sputum may present a significant challenge for the development of effective inhalable ON formulations [13].

An approach recently proposed to overcome airway barriers and transfect differentiated respiratory epithelial cells with nucleic acids, relies on the use of cationic polymers [14]. In particular, polyethylenimine (PEI) is gaining increasing research interest in pulmonary delivery of nucleic acids to improve ON transfection efficiency toward respiratory epithelial cells [15]. The use of PEI in treatment of chronic lung diseases could be even more interesting if one considers that PEI carries antibacterial properties toward Gram (-) bacteria, such as *P. aeruginosa*, both *in vitro* [16], [17] and *in vivo* [18]. Furthermore, PEI may behave as a mucoactive agent due to its well-established osmotic nature and allow favorable fluidification of lung secretions, which *a priori* can be beneficial to promote ON transport toward epithelium. Thus, once biocompatibility issues are sufficiently addressed, pulmonary delivery of PEI along with decoy ONs against NF-κB may become an attractive therapeutic strategy for the treatment of complex lung pathologies (i.e., CF) requiring a multidrug approach aimed at controlling chronic inflammation, infection and iperproduction of a viscous mucus [19].

As a biodegradable polymer, poly(lactic-co-glycolic acid) (PLGA), which is already approved for human use by parenteral injection, can be of great help in developing novel inhalable formulations for biotech drugs, such as decoy ONs [20]. If adequately engineered, PLGA carriers may allow intact ONs to gain access to the target cells at the right time and for proper duration. Along these lines, we have developed biodegradable large porous particles (LPP) for local and prolonged delivery of a decoy ON to NF-κB in the lung (dec-ODN), made of PLGA and a lipid helper excipient, namely 1,2-dipalmitoyl-sn-glycero-3-phosphocholine (DPPC) [21], [22]. Actually, LPP can be engineered into dry powders [22], which are recently emerging as a formulation of choice for macromolecule delivery to the lung. Furthermore, LPP geometric and mass mean aerodynamic diameters can be tuned to attain a widespread deposition in the deep lung as well as macrophage escape [21]–[23]. Finally, LPP cause a prolonged inhibitory effect of dec-ODN on NF-κB/DNA binding activity and related IL-6 and IL-8 expression in LPS-stimulated CF human bronchial epithelial cells [21].

Taking for granted the beneficial properties of LPP developed in our previous studies, here we try to add “adjuvant” functionality by engineering LPP containing dec-ODN with PEI. We investigate how PEI addition in LPP may affect those carrier properties considered crucial to the development of therapeutically-relevant delivery systems. In analogy to our previous findings, the effect of dec-ODN released from PEI/PLGA composite LPP (LPP_PEI_) on the expression of IL-8 in LPS-stimulated CF human bronchial epithelial IB3-1 cells was assessed. Afterwards, taking into account that mucin gene expression in lung disease is transcriptionally and post-transcriptionally regulated by inflammatory mediators [24] and *P. aeruginosa* LPS induces the expression of MUC2 gene *via* NF-κB [24], [25], we studied the effects of LPP_PEI_ on MUC2 gene expression in LPS-stimulated mucoepidermoid carcinoma cells (NCI-H292).

## Results

### Overall Properties of the Developed LPP

**Figure 1:**
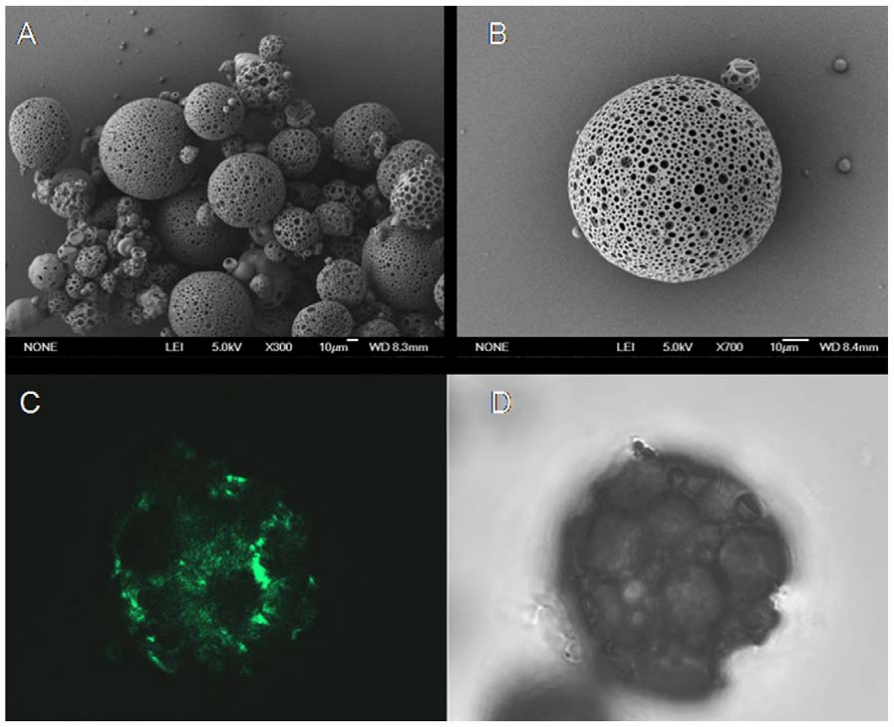
Morphological analysis of LPP_PEI_ containing dec-ODN. (A–B) SEM micrographs at different magnifications; (C) confocal microscopy section (PLGA-FITC in green) and light microscopy images of FITC-labeled LPP_PEI_. Field is representative of the formulation.

Biodegradable PLGA-based LPP containing dec-ODN were prepared with good yields by the double emulsion technique employing ammonium bicarbonate as porogen. SEM analysis performed on a representative LPP_PEI_ sample showed that the adopted formulation conditions allowed the achievement of a homogeneous population of spherical and porous particles (Figure 1A). As can be seen in figure 1B, surface pores appear small, regular, and uniformly distributed throughout the polymeric matrix. CLSM analysis of fluorescent LPP_PEI_ confirmed the internal macroporous structure of the developed formulations (Figure 1C).

The overall properties of LPP_PEI_ are reported in table 1. By a similar tapped density (the tapped density of LPP_DPPC_ was found to be around 0.035 g/ml), LPP_PEI_ displayed a volume mean diameter significantly higher than the corresponding LPP_DPPC_ (≈31.5 µm) [21]. As a consequence, a higher MMAD_t_ was estimated for LPP_PEI_ as compared to LPP_DPPC_ (≈6 µm). Anyway, MSLI tests demonstrated that the developed LPP_PEI_ had very good aerosolization properties, with more than 95% of the capsule content being emitted during aerosolization, very high FPF and an experimental MMAD<4 µm. Concerning LPP entrapment efficiency, about 70% and 40% of the total amount of dec-ODN and PEI respectively added to the formulation were effectively entrapped within LPP_PEI_.

**Table 1:** Overall properties of LPP_PEI_ containing dec-ODN.

	LPP_PEI_
Volume mean diameter (d)^1^ (**µ**m±S.E.M.^2^)	45.9±3.3
Tapped density (*ρ*) (g/ml ±S.E.M.^2^)	0.043±0.004
MMAD_t_ 3 (**µ**m±S.E.M.^2^)	9.5±0.4
Emitted dose (EM) (%±S.E.M. 2)	99.7±8.3
Fine Particle Fraction (FPF) (%±S.E.M. 2)	69.2±2.6
MMAD_exp_ (**µ**µ±GSD)^4^	3.6±1.5
dec-ODN actual loading^5^ (nmol/mg±S.E.M.^2^)	0.087±0.005
dec-ODN encapsulation efficiency^5^ (%±S.E.M.^2^)	65.6±3.6
PEI encapsulation efficiency^6^ (%±S.E.M.^2^)	36.5±3.8

1 Mean geometric diameter as determined by laser diffraction.

2 Standard Error Mean calculated on 3 different batches (n = 6).

3 Theoretical mass mean aerodynamic diameter estimated on the basis of the equation 2.

4 Experimental mass mean aerodynamic diameter ± geometrical standard deviation as evaluated by MSLI tests.

5 Percent ratio between dec-ODN actual and theoretical loadings. Dec-ODN theoretical loading was 0.140 nanomoles *per* mg of LPP_PEI_.

6 Percent ratio between PEI actual and theoretical loadings. PEI theoretical loading was 1.0 mg *per* 100 mg of LPP_PEI_.

**Figure 2:**
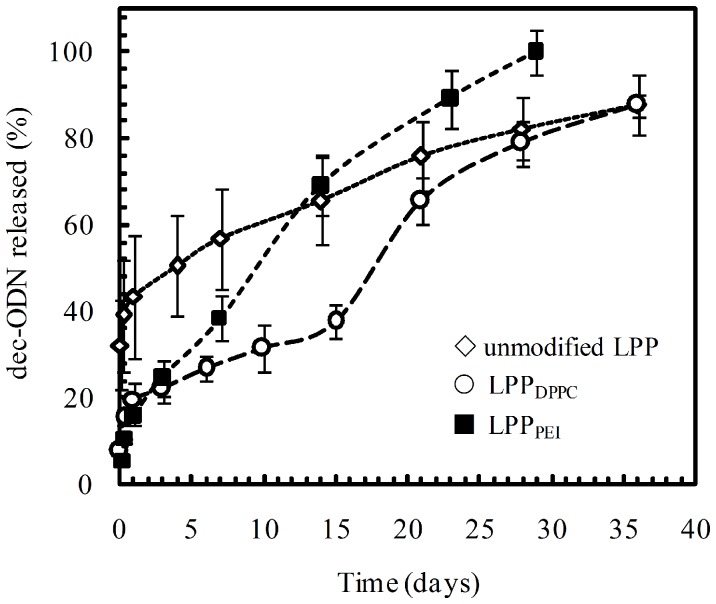
*In vitro* release profile of dec-ODN from the developed LPP at pH 7.2 and 37°C. Data are expressed as mean ± S.E.M. of two experiments performed in triplicate (n = 6).

Results of *in vitro* release studies are reported in Figure 2 as the percentage of dec-ODN released over time. In each case, a controlled release of dec-ODN from LPP lasting for 30 days was achieved. LPP prepared without helper excipients (i.e., unmodified LPP) showed a biphasic release profile and released about 40% of their content in the first 6 h (i.e., burst).

On the contrary, LPP_DPPC_ displayed a triphasic release profile of dec-ODN, characterized by low burst (8±2% of dec-ODN released after 6 h), followed by a slower release phase lasting about 15 days and a final rapid release of dec-ODN. Finally, the release of dec-ODN from LPP_PEI_ was a two-stage process where a burst as low as 5±1% was followed by a continuous release of dec-ODN approximating a zero-order kinetics. We have also tried to assess LPP release kinetics from simulated CF mucus in simulated interstitial lung fluids. Nevertheless, to avoid the interference of CF mucus components on the quantitation of dec-ODN, it was necessary to modify LPP formulation by increasing dec-ODN actual loading. Noteworthy, no significant difference in dec-ODN release from modified LPP was observed in simulated lung fluids as compared to results achieved in phosphate buffer, suggesting that CF mucus components did not affect dec-ODN release from the developed LPP (data not shown).

### Stability of Dec-ODN Entrapped in LPP_PEI_

**Figure 3:**
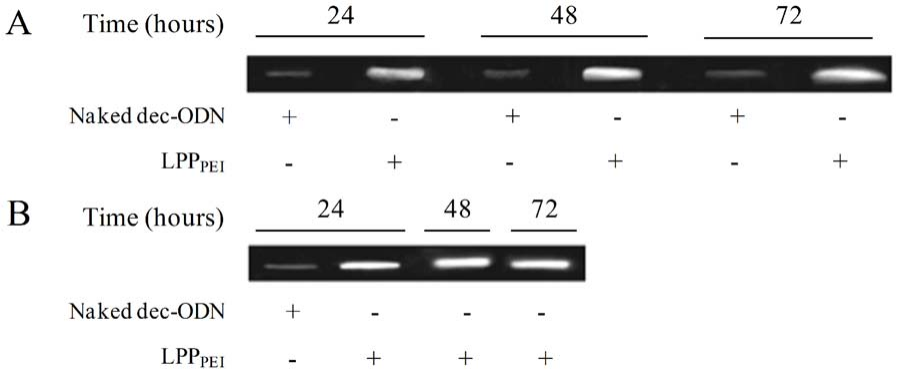
Agarose gel electrophoresis of LPP_PEI_ or naked dec-ODN collected from cell culture medium at 24, 48 and 72 h. (A) dec-ODN released from LPP_PEI_ or naked dec-ODN from supernatant; (B) dec-ODN extracted from LPP_PEI_ pellet and annealed naked dec-ODN (internal control). Data are from a single experiment and are representative of three separate experiments.

The stability of dec-ODN entrapped in LPP_PEI_ and naked dec-ODN in NCI-H292 cell culture medium collected at 24, 48 and 72 h was assessed by gel electrophoresis. As shown in Figure 3A, dec-ODN released from LPP_PEI_ at 24 h exhibited a marked band which intensity was reduced at 48 and 72 h, indicating a slow release of dec-ODN from LPP_PEI_. In contrast, the band of naked dec-ODN was evident at 24 h and smeared at 48 and 72 h, suggestive of its partial degradation. Furthermore, the band of dec-ODN extracted from LPP_PEI_ pellet (Figure 3B) was observed at all time points considered, showing a long-lasting protection of dec-ODN by LPP_PEI_. Similar results were observed in IB3-1 cells incubated with LPP_DPPC_ (Figure S1, Supporting Information S1).

### In vitro Cytotoxicity of LPP_PEI_

**Figure 4:**
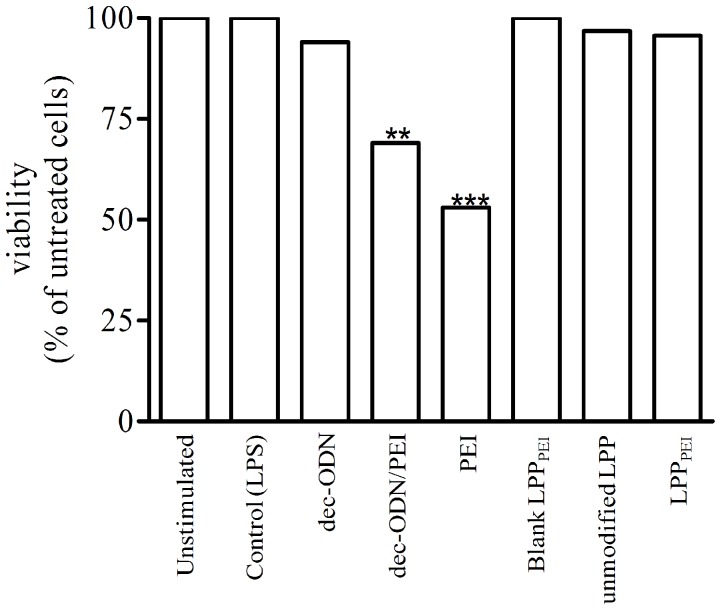
MTT assay shows the effect of PEI alone, naked dec-ODN/PEI complexes or LPP_PEI_ on NCI-H292 cell viability. Data are mean±S.E.M. of three experiments in triplicate. **p<0.001, ***p<0.001 *vs*. unstimulated cells.

Since an important concern in using PEI relates to its toxicity profile, we investigated the effects of PEI alone, naked dec-ODN/PEI or both entrapped in LPP_PEI_ on NCI-H292 cell viability at 24 h. As can be seen in figure 4, PEI alone or naked dec-ODN/PEI tested at the same levels present in LPP_PEI_ significantly reduced cell viability, whereas LPP_PEI_ did not affect the number of viable cells. Similar results were achieved on IB3-1 cells (data not shown).

### Effect of LPP_PEI_ on IL-8 mRNA Expression in CF Bronchial Cells

**Figure 5:**
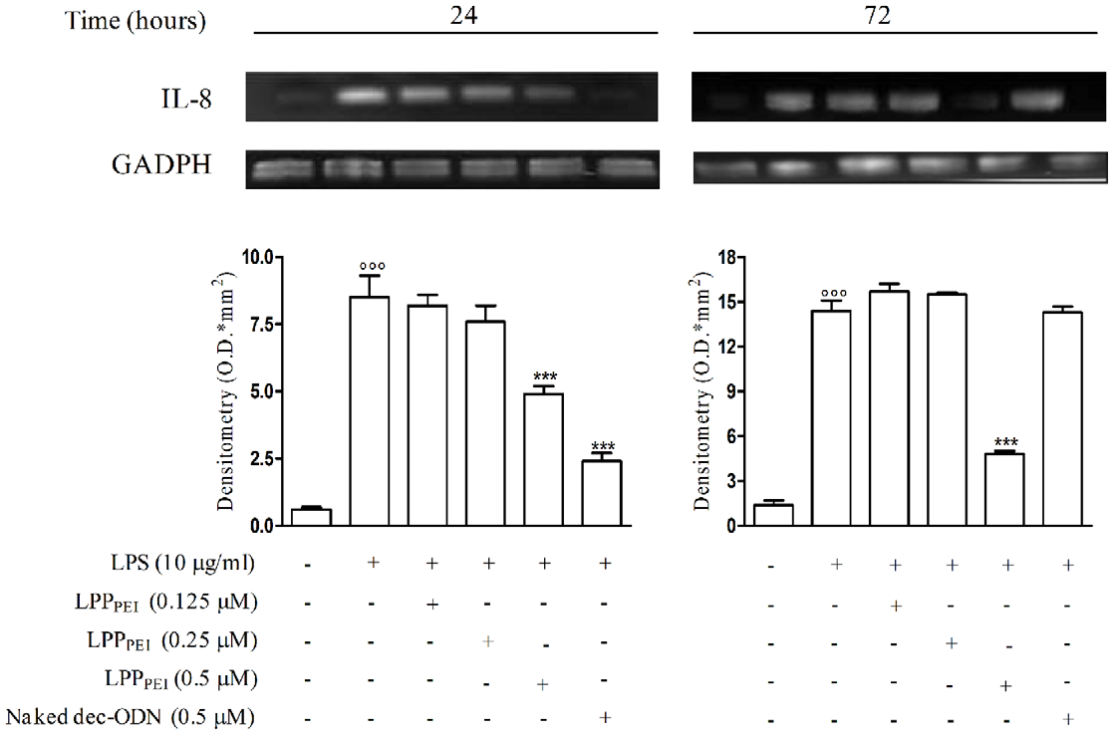
Representative RT-PCR and corresponding densitometric analysis show the effects of LPP_PEI_ on IL-8 mRNA levels induced by LPS in IB3-1 cells at 24 and 72 h. Densitometric data are expressed as mean±S.E.M. of three experiments. °°°p<0.001 *vs*. unstimulated cells; ***p<0.001 *vs*. LPS.

We first investigated the effects of LPP_PEI_ on IL-8 mRNA expression in ΔF508 CFTR-mutated human bronchial epithelial IB3-1 cells stimulated with LPS from *P. aeruginosa.* LPS challenge of cells for 24 and 72 h induced a significant increase in the IL-8 mRNA levels, as compared to unstimulated cells. Treatment of cells with LPP_PEI_ at 0.125 and 0.250 µM did not cause any effect, whereas 0.5 µM LPP_PEI_ significantly prevented IL-8 mRNA expression (by 42.35±0.3%, 40.27±0.3% at 24, and 72 h, respectively; *n* = 3). Notably, when used at the same concentration, naked dec-ODN prevented IL-8 mRNA expression only at 24 h (by 71.76±0.3%; *n* = 3) (Figure 5). A comparable profile was observed on IL-6 and IL-8 protein levels in the culture medium of the same cells incubated with dec-ODN released by LPP_DPPC_ (Figure S2, Supporting information S1). Cell viability was not affected by any treatment (≥85%).

### Effect of LPP_PEI_ on NF-κB/DNA Binding Activity Activity

**Figure 6:**
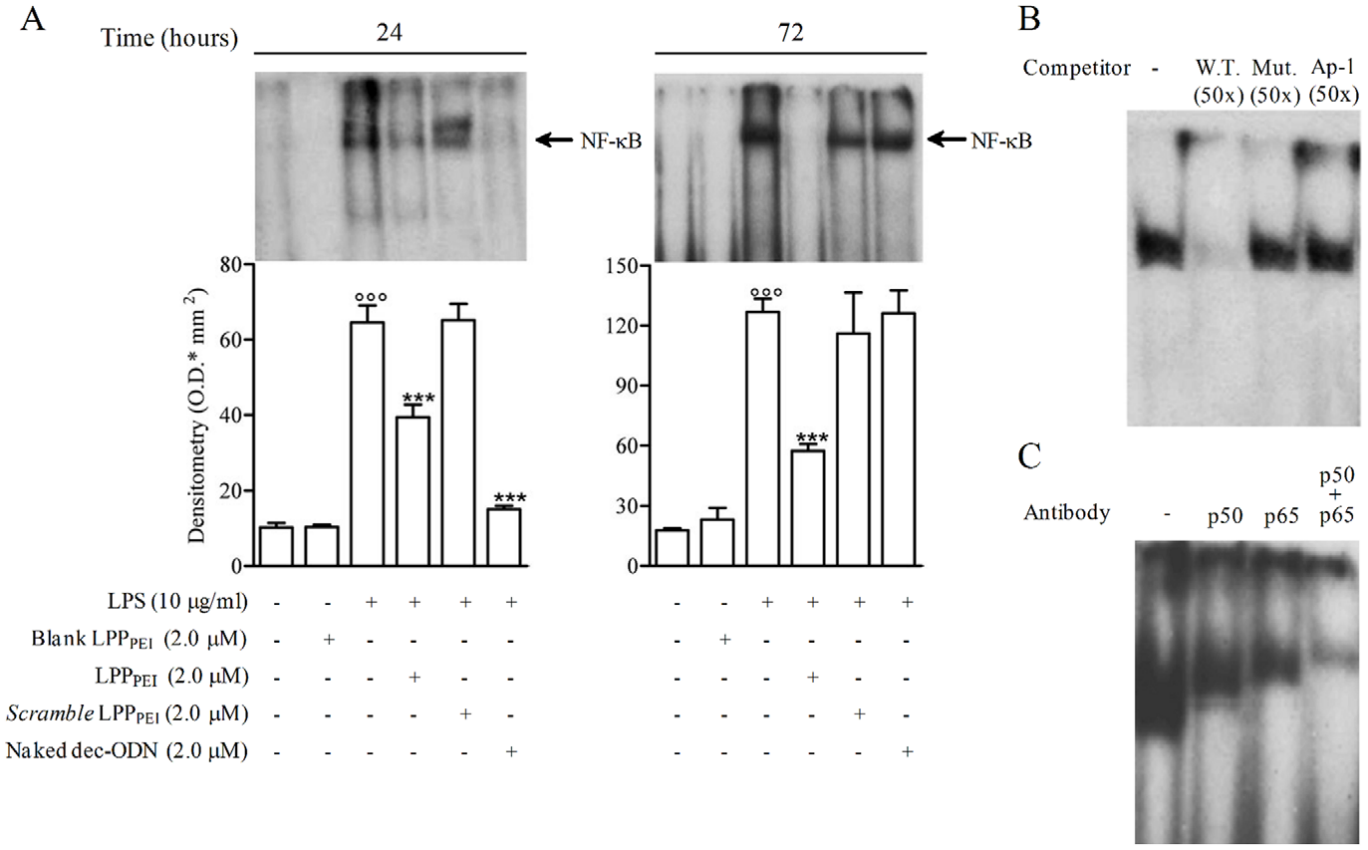
Representative EMSA and corresponding densitometric analysis show the effects of dec-ODN released from LPP_PEI_ or naked dec-ODN decoy on NF-κB/DNA binding activity in LPS-stimulated NCI-H292 cells at 24 and 72 h. Densitometric data are expressed as mean ± S.E.M. of three separate experiments. °°°p<0.001 vs. unstimulated cells; ***p<0.001 vs. LPS alone. (B-C) Characterization of NF-κB/DNA complex was performed on nuclear extracts from LPS-stimulated NCI-H292 cells at 24 h (W.T. = *wild tipe* dec-ODN; Mut. = *scramble* dec-ODN). Plots in (A,B,C) are from a single experiment and are representative of three separate experiments.

The effect of dec-ODN released from LPP_PEI_ or naked dec-ODN on NF-κB/DNA binding activity was evaluated on nuclear extracts from LPS-stimulated NCI-H292 cells for 24 and 72 h by EMSA. LPS challenge (10 µg/ml) induced a significant increase of DNA/protein complexes, as compared to untreated cells. Incubation of cells with LPP_DPPC_ did not cause any effect on NF-κB/DNA binding activity and MUC2 mRNA levels even at concentrations 4 folds higher than those used in IB3-1 cells (LPP_DPPC_ 0.5 µM) (data not shown). Contrariwise, incubation of cells with LPP_PEI_ (2.0 µM) resulted in a significant reduction of NF-κB/DNA binding activity at 24 and 72 h (38.92±3.37% and 54.71±3.36%, respectively; *n = 3*) (figure 6A). Naked dec-ODN, at the same concentration, inhibited NF-κB/DNA binding activity only at 24 h (76.64±0.92%; *n = 3*). *Scramble* dec-ODN released from LPP_PEI_ (*Scramble* LPP_PEI_) as well as blank LPP did not exhibit any effect (Figure 6A).

The composition of the NF-κB complex was determined by competition and supershift experiments in nuclear extracts from LPS-stimulated NCI-H292 cells (Figure 6B and 6C). The specificity of the NF-κB/DNA binding complexes was evident by the complete displacement of the binding in the presence of a 50-fold molar excess of unlabelled NF-κB probe in the competition reaction (figure 6B). In contrast, a 50-fold molar excess of unlabelled mutated NF-κB probe or Ap-1 oligonucleotide had no effect on DNA-binding activity. The composition of the NF-κB complex activated by LPS was determined by using specific antibodies against p50 and p65 subunits of NF-κB (figure 6C). The anti-p50 and anti-p65 antibodies clearly gave rise to a characteristic supershift of the retarded complex, suggesting that the NF-κB complex contained p50 and p65 heterodimers.

**Figure 7:**
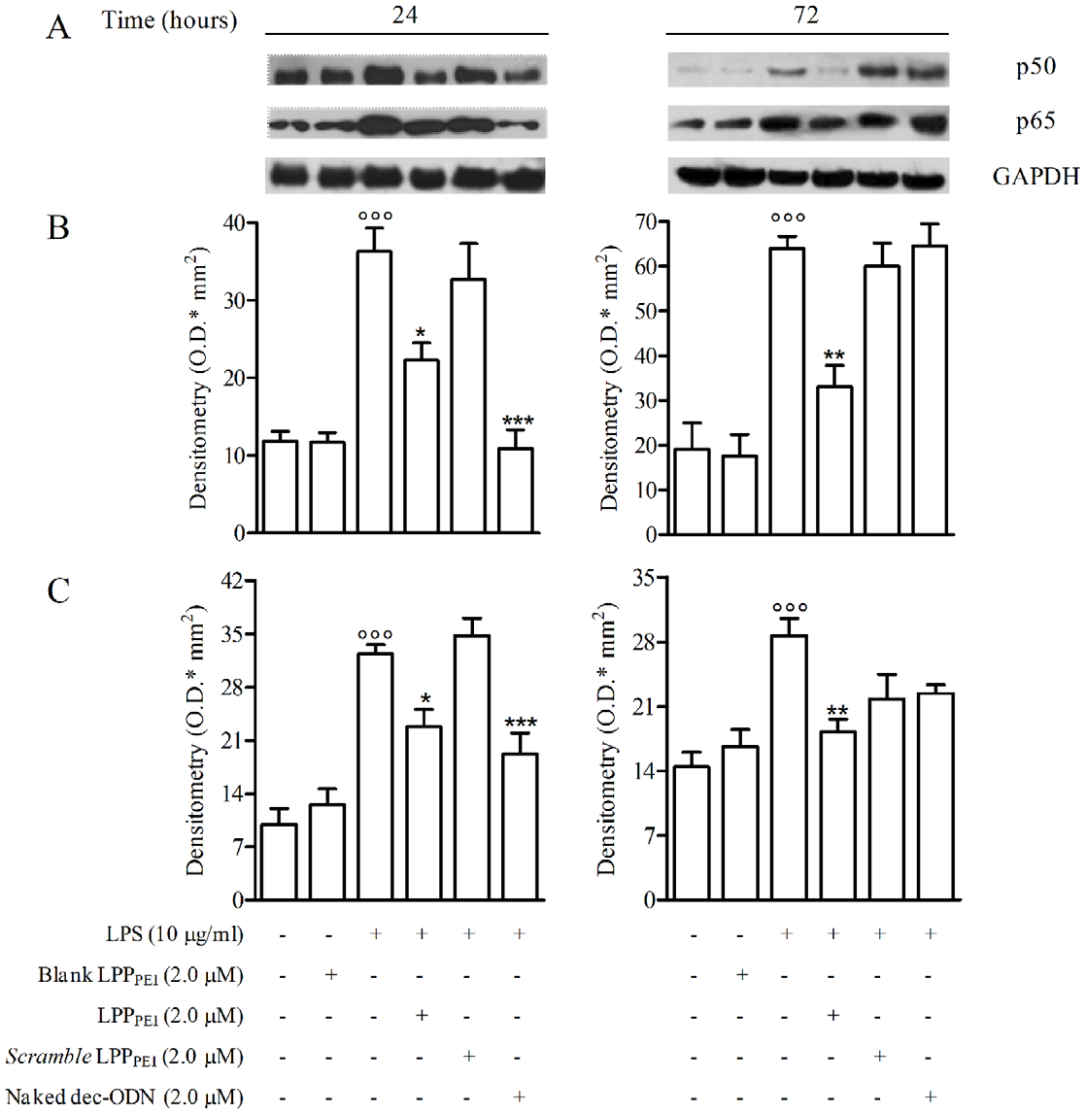
Representative Western blot and corresponding densitometric analysis show the effects dec-ODN released from LPP_PEI_ or naked dec-ODN on p50 and p65 nuclear translocation in LPS-stimulated NCI-H292 cells at 24 and 72 h. GAPDH is reported as a control. Densitometric data in B (p50) and C (p65) are expressed as mean ± S.E.M. of three separate experiments. °°°p<0.001 *vs.* unstimulated cells; *p<0.05, ***p<0.001 *vs.* LPS alone. Data in (A) are from a single experiment and are representative of three separate experiments.

The effect of dec-ODN released from LPP_PEI_ or naked dec-ODN on NF-κB/DNA binding activity was confirmed by evaluating nuclear levels of p50 and p65 by Western blot (figure 7A).

Stimulation of cells with LPS induced a significant increase of p50 and p65 nuclear translocation, as compared to untreated cells at 24 and 72 h. Treatment of cells with LPP_PEI_ (2.0 µM) caused, at 24 (Figure 7B) and 72 h (Figure 7C), a significant reduction of nuclear levels of p50 (38.54±2.23 and 48.35±4.79 respectively; *n = 3*) and p65 (29.59±2.25 e 36.46±1.39, respectively; *n = 3*). Naked dec-ODN, at the same concentration, reduced p50 and p65 nuclear translocation only at 24 h (70.03±2.46 and 40.38±2.77, respectively; *n = 3*). LPP_PEI_ containing *scramble* dec-ODN (*scramble* LPP_PEI_) as well as blank LPP_PEI_ did not exhibit any effect (figure 7). All the treatments, alone or in combinations, did not affect cell viability (>95%).

### Effect of LPP_PEI_ on MUC2 Expression

**Figure 8:**
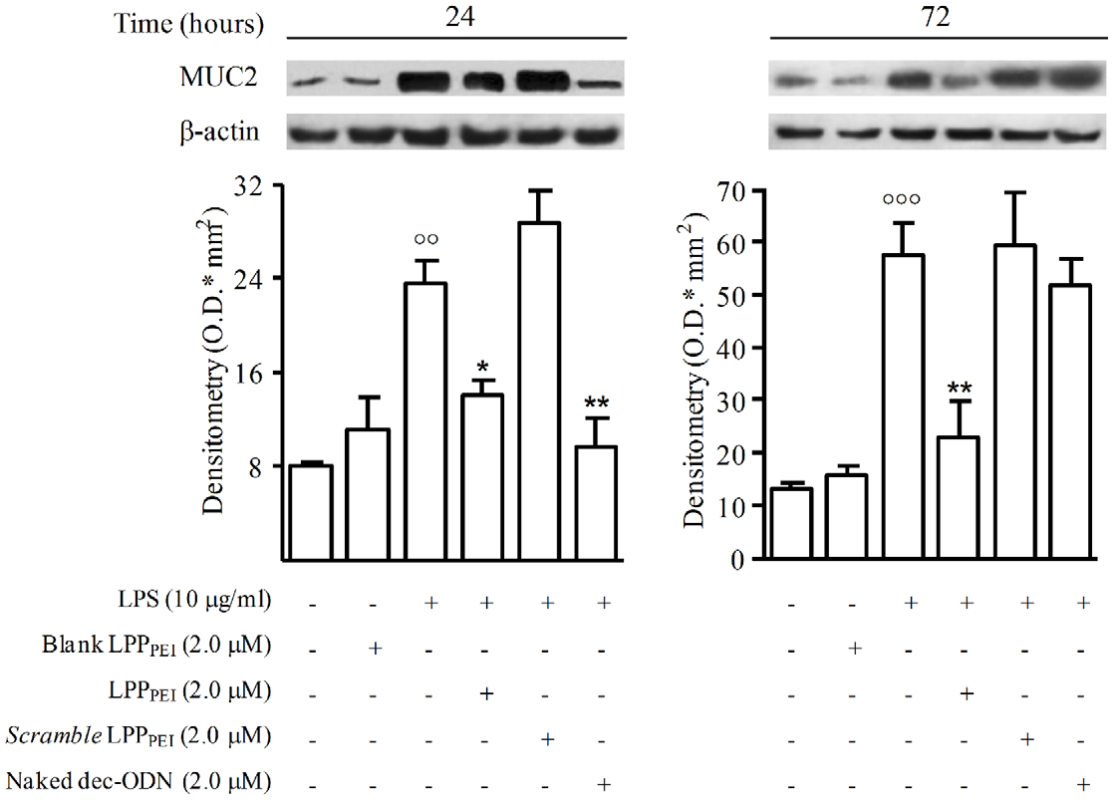
Representative Western blot and corresponding densitometric analysis show the effects of dec-ODN released from LPP_PEI_ or naked dec-ODN on MUC2 expression in LPS-stimulated NCI-H292 cells at 24 and 72 h. β-actin is reported as a control. Densitometric data are expressed as mean ± S.E.M. of three separate experiments. °°p<0.01, °°°p<0.001 *vs.* unstimulated cells; *p<0.05, ***p<0.001 *vs.* LPS alone. Immunoblots are from a single experiment and are representative of three separate experiments.

The expression of MUC2 protein was significantly augmented in cytoplasmic extracts from NCI-H292 cells stimulated with LPS (10 µg) at 24 and 72 h, as compared to untreated cells. As observed for NF-κB transcriptional activity, incubation of cells with LPP_DPPC_ did not cause any effect on MUC2 expression (data not shown). Contrariwise, incubation of cells with LPP_PEI_ (2.0 µM) significantly reduced MUC2 protein expression at 24 and 72 h (40.44±1.28 and 59.78±6.69, respectively; *n* = 3). Naked dec-ODN, at the same concentration, exhibited this effect only at 24 h (58.92±2.41; *n* = 3). *Scramble* dec-ODN released from LPP_PEI_ (*scramble* LPP_PEI_) as well as blank LPP_PEI_ did not exhibit any effect (Figure 8).

**Figure 9:**
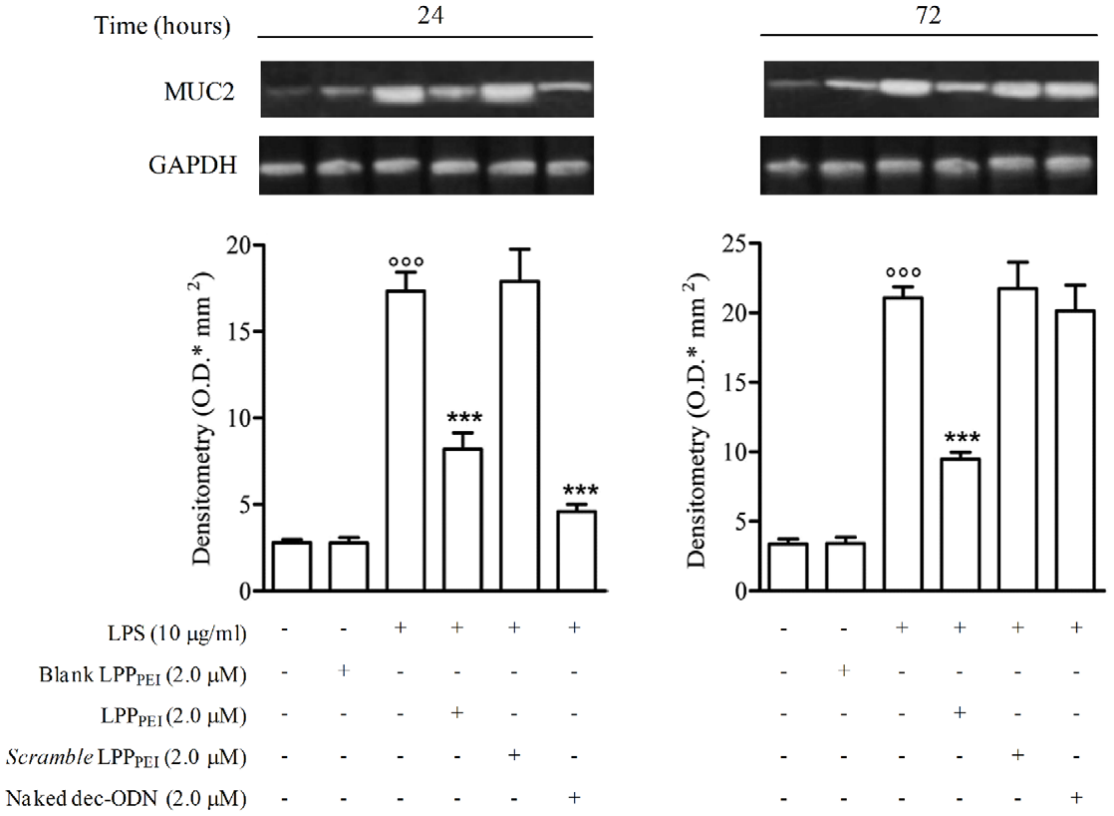
Representative RT-PCR and corresponding densitometric analysis show MUC2 mRNA levels induced by LPS in NCI-H292 cells at 24 and 72 h. GAPDH mRNA levels are reported as a control. PCR plots are from a single experiment and are representative of three separate experiments. Densitometric data are expressed as the mean ± S.E.M. of three experiments. °°°p<0.001 *vs.* unstimulated cells; ***p<0.001 *vs.* LPS.

To assess whether the decrease in MUC2 protein expression was related to a reduced MUC2 gene expression, we analyzed MUC2 mRNA levels by RT-PCR (figure 9). Stimulation of NCI-H292 cells with LPS (10 µg/ml) for 24 and 72 h determined a significant increase of MUC2 mRNA levels, as compared to untreated cells. Incubation of cells with LPP_PEI_ (2.0 µM) inhibited in a significant manner MUC2 mRNA levels (52.70±0.95% and 55.05±0.49% at 24 and 72 h, respectively; *n = 3*). Naked dec-ODN, at the same concentration, reduced MUC2 expression only at 24 h (73.50±0.40%; *n = 3*). *Scramble* dec-ODN released from LPP_PEI_ as well as blank LPP_PEI_ did not exhibit any effect.

## Discussion

The translation of oligonucleotide (ON)-based respiratory therapies in humans runs into the need of specifically tailored inhalable formulations able to: i) protect the entrapped ON from *in vitro/in vivo* degradation; ii) promote ON transport through lung mucus barriers; iii) facilitate ON uptake by lung epithelial cells, where its target is located. In this scenario, we are developing large porous particles (LPP) for pulmonary delivery, focusing on a decoy oligodeoxynucleotide against NF-κB (dec-ODN) [21]. These powders represent the first example of dry powders engineered for ON inhalation. We have already evidences that the developed LPP (i.e., LPP_DPPC_) may cause a prolonged inhibitory effect of dec-ODN on NF-κB/DNA binding activity and related IL-6 and IL-8 expression in LPS-stimulated CF cells [21]. We confirm here the ability of the previously developed LPP_DPPC_ in inhibiting IL-6 and chemoattractant IL-8 secretion from ΔF508 CFTR-mutated human bronchial epithelial cells stimulated with LPS from *P. aeruginosa*, as shown in Supporting information S1 (figure S1). In the attempt to further enhance carrier properties and anti-inflammatory effects of previously developed LPP (i.e., LPP_DPPC_), PLGA/PEI composite LPP have been developed and their effects on human airway epithelial cells stimulated with LPS were evaluated. In fact, PEI could represent an interesting candidate for lung delivery of ONs in the treatment of chronic lung disease, such as CF, owing to its proved antibacterial properties toward Gram (-) bacteria and potential mucoactive effects.

Since LPP_PEI_ are conceived for local and prolonged dec-ODN release in the lung, some crucial technological aspects of the particles were taken into account: primary bulk properties (e.g., morphology, density and size), encapsulation efficiency and *in vitro* dec-ODN release profile. In term of size and density, large but “light” particles (i.e., mass density lower than 0.4 g/cm^3^) may represent a useful mean to reach more distal airways and avoid natural macrophage-mediated lung clearance mechanisms ensuring that encapsulated dec-ODN is effectively delivered to lung epithelium. We have found that processing of PLGA/PEI mixtures in the presence of ammonium bicarbonate may allow the production of gas-foamed LPP_PEI_ displaying large size and a highly porous structure. Pore formation, due to gas bubbles evolution during LPP_PEI_ preparation, was well controlled in analogy to what found for previously developed LPP_DPPC_. LPP_PEI_ showed a higher geometric diameter as compared to LPP_DPPC_, likely ascribable to PEI partitioning in the internal water phase of the double emulsion. The osmotically active ingredient may generate an osmotic pressure gradient, allowing the transport of water across the organic phase. As previously observed, the occurrence of this process during particle hardening results not only in a higher particle porosity but also in a potential increase of particle dimensions [26]. The estimate of MMAD_t_ suggests good aerodynamic properties for LPP_PEI_ with values lower than 10 µm, 4.5-fold lower than the corresponding geometric mean diameter. Nonetheless, if particle porosity is essential to achieve respirable particles, this can be detrimental for ON entrapment efficiency and release profile. Furthermore, particles that are “too porous” may easily imbibe extracellular fluids, being reach of nucleases, and the encapsulated ON is expected to be more exposed in such loose polymer matrices.

Release rate is a critical factor to exert a temporal control over the administered ON dose. Notably, the approach of using PEI to optimize powder properties is further strengthened by the results obtained in this work. Unmodified LPP prepared without helper excipients show a poorly modulated biphasic release of the encapsulated ON, which is typical for porous PLGA particles [27], [28]. Contrariwise, LPP_PEI_ provide not only an effective encapsulation of dec-ODN within LPP, but also its sustained release *in vitro* for more than 30 days. Notably, the release profile of dec-ODN depended upon the adopted helper excipient and was approximately of zero-order in case of LPP_PEI_. During the diffusion-erosion phase, dec-ODN molecules located inside LPP macropores will naturally diffuse towards one of the endpoints of the pore and, where pores are interconnected, reach release medium [29], [30]. Thus, dec-ODN slow release from modified LPP can be reasonably attributed to poor pore interconnection, meaning that dec-ODN may be released only when micropores evolve, increasing space available for diffusion. Nonetheless, in case of LPP_PEI_ the contribution of the electrostatic interactions occurring between dec-ODN and PEI on release rate plays an important role. Actually, dec-ODN is negatively-charged and likely able to interact with the polycation at the interfaces, where PEI is thought to be preferentially located [31]. Thus, these results confirm the importance of the helper excipient to modulate release of dec-ODN from gas-foamed LPP and, in general, to employ PEI when LPP are conceived for lung delivery of nucleic acids. Moreover, the developed LPP formulations increased the stability of dec-ODN in cell culture medium up to 72 h. To our knowledge, this is the first time that highly porous PLGA particles are proven to be as effective as their nonporous counterparts in preserving the integrity of the encapsulated macromolecule in cultured cells.

On the basis of the encouraging technological premises, we evaluated the effects of LPP_PEI_ on the expression on IL8 and MUC2 expression in human airway epithelial cells stimulated with LPS. Several studies showed that PEI may itself stimulate the immune system and cause various degrees of pro-inflammatory cytokine secretion [15], [32], [33]. Here, we show that cell exposure to LPP_PEI_ does not induce cytotoxic effects in all the cell lines tested whereas free PEI or PEI/dec-ODN significantly reduce cell viability. These results likely suggest that the use of PEI in PLGA formulations able to exert a control over PEI dose administered to the cells in time may result in a lower toxicity as compared to its bolus administration. Actually, recent *in vitro* toxicological data suggest that when PEI is used within a therapeutically relevant concentration range either as polyplex or free, no cytotoxicity and only minor immunomodulatory responses are elicited on murine alveolar epithelial–like type II cells and murine alveolar macrophages [33].

In analogy to previously developed LPP_DPPC_, LPP_PEI_ cause a prolonged inhibition of IL-8 expression in LPS-stimulated IB3-1 cells up to 72 h, whereas naked dec-ODN exhibits these effects only at 24 h. The fact that LPP_DPPC_ and LPP_PEI_ work by a similar extent likely suggest that endocytosis mediated by caveolin, already reported to be abundantly expressed in lung tissue including IB3-1 cells [34], [35], may be the preferential route of cell internalization of dec-ODN released from LPP. Actually, caveolar vescicles and caveosomes have a neutral pH, where the buffering capacity of PEI, the so called “proton sponge” nature of the polycation, is not required to protect the ON from acidic endosomal compartment and, in so doing, achieve its more efficient delivery to the target [36].

Very interesting results were obtained on LPP_PEI_ in human lung mucoepidermoid carcinoma cells (NCI-H292), a well-established *in vitro* model for investigating MUC gene expression in airway diseases [6], [37]. Although the presence of MUC2 glycoprotein in normal mucus or CF sputum has not been demonstrated yet, there is evidence that MUC2 gene expression is upregulated in CF airways [7]. Both bacteria (e.g., *P. aeruginosa*) and several cytokines/chemokines relevant to CF have been reported to transcriptionally upregulate MUC2 gene expression [6]–[9], thus contributing to the production of the thick and tenacious sputum of CF patient [13]. This is not only determinant for progressive lung destruction and irreversible respiratory failure but represents also the primary obstacle to ON diffusion and interaction with underlying epithelial cells. Here, NCI-H292 cells stimulated with LPS from *P. aeruginosa* caused a significant increase of NF-κB/DNA binding activity as well as MUC2 protein and mRNA expression. Noteworthy, cell exposure to LPP_DPPC_ did not result in any inhibitory effect, even at concentrations 4-folds higher than those tested on CF cells. By contrast, due to the presence of PEI and dec-ODN slow release, LPP_PEI_ showed a persistent inhibition of NF-κB related MUC2 gene expression at concentrations much lower as compared to naked dec-ODN. These results suggest that different mechanisms control internalization of dec-ODN in mucoepidermoid cells. Thus, engineering LPP with PEI may play a crucial role in controlling mucin gene expression and, in turn, become a key factor for *in vivo* translation of dec-ODN therapy.

Taken all together, our findings suggest that the developed LPP are respirable upon delivery from a breath-actuated DPI, exert a temporal control over dec-ODN released amounts, while preserving its integrity, and cause a persistent inhibition of IL-8 and MUC2 expression as compared to naked dec-ODN. LPP_PEI_ features could all contribute to better control local chronic inflammation and prompt us to go in depth into their *in vivo* safety and potential. To date, the safety and effectiveness of decoy ONs against NF-κB to prevent restenosis has been assessed in an open-label phase I/IIa clinical trial and no significant systemic adverse effect occurred in any of the patients along six months of observation [38]. However, the side effects related to the use of decoy ONs against NF-κB in humans are not fully elucidated yet. Pulmonary delivery should present the advantage to selectively target the interested areas of the respiratory tract without the drawback of dec-ODN release to other non-target organs and tissues. Of course, further studies first in reliable animal models and then in clinics must be performed to assess the safety and potential of the proposed approach.

### Conclusions

Dry powders for dec-ODN delivery in the lung were successfully engineered to provide a new therapeutic strategy for inhibiting NF-κB transcriptional activity and its related gene expression in chronic lung inflammation of CF patients. Formulation of dec-ODN in respirable particles based on combinations of PLGA and PEI allowed its protection in biologically relevant conditions and sustained release. Our findings demonstrate a persistent inhibition of IL-8 and MUC2 expression in LPS-stimulated airway epithelial cells and highlight the role of PEI in enhancing carrier properties while suppressing its toxicity. These promising indications prompt us to go in depth into the potential of respirable LPP_PEI_ in CF treatment, with particular regard to further roles of PEI as multipurpose agent.

## Materials and Methods

### Materials

Phosphorothioate oligodeoxynucleotide synthesis was performed by Tib Molbiol (Roche Diagnostics, Italy). Poly(D,L-lactide-co-glycolide) (50∶50) (PLGA) (Resomer RG 504 H; Mw 41.9 kDa; inherent viscosity 0.5 dl/g) was purchased from Boehringer Ingelheim (Germany). Polysorbate 80, polyvinylalcohol (PVA, Mowiol® 40–88), sodium azide, 1,2-dipalmitoyl-*sn*-glycero-3-phosphocholine (DPPC), and ammonium hydrogen carbonate were obtained from Sigma-Aldrich (Italy). Analytical grade sodium chloride, potassium chloride, sodium phosphate dibasic anhydrous, sodium bicarbonate, methylene chloride, were supplied by Carlo Erba (Italy).

### Transcription Factor Decoy Oligodeoxynucleotide

A plain double-stranded decoy oligodeoxynucleotide to NF-κB (dec-ODN) and its corresponding *scramble* dec-ODN were prepared by annealing sense and antisense phosphorothioate ODNs *in vitro* in filtered water solution. In order to denature any secondary structure and to pair sense and antisense nucleotide sequences by hydrogen bonds, the mixture was heated at 80°C for 5 min and allowed to cool slowly at room temperature over 18 h. For each experiment, dec-ODN and *scramble* dec-ODN were annealed before use.

The wild-type NF-κB consensus sequence used (dec-ODN) was:

5′-GAT CGA GG**G GAC** TTT CCC TAG C-3′

3′-CTA GCT CC**C CTG** AAA GGG ATC G-5′

Mutant NF-κB consensus sequence with a mutation of the bases written in bold (GGAC to AAGC) of the wild-type NF-κB consensus sequence (*scramble* dec-ODN) was used:

5′-GAT CGA GG**A AGC** TTT CCC TAG C-3′

3′-CTA GCT CC**T TCG** AAA GGG ATC G-5′

### Particle Production and Characterization

PLGA-based large porous particles containing dec-ODN were prepared by a modified double emulsion-solvent evaporation technique assisted by gas-foaming [22]. Briefly, 0.25 ml of water containing (NH_4_)HCO_3_ at 10% (w/v) and 0.6 mg of dec-ODN were poured into 2.5 ml of methylene chloride containing 375 mg of PLGA. DPPC at 0.1% (w/v) or PEI (PEI/PLGA 1∶100 w/w) were added to the PLGA-containing organic phase, so as to achieve LPP_DPPC_ and LPP_PEI_ dec-ODN formulations, respectively. Emulsification was achieved using a high-speed homogenizer (Ystral, Heidolph, Germany) operating at 755 *g* (tool 6G) for 3 minutes. The primary w/o emulsion was rapidly added to 25 ml of a 0.5% (w/v) PVA (Mowiol® 40–88) aqueous solution and homogenised at 676×*g* (tool 10F) for 2 minutes to produce the multiple emulsion. Solvent evaporation and subsequent particle hardening was achieved under magnetic stirring (MR 3001K, Heidolph, Germany) at room temperature. After 3 h, particles were collected, washed three times with distilled water by centrifugation (Hettich Zentrifugen, Universal 16R) at 4°C and frozen in liquid nitrogen. Samples were then dried for 36 h by a Modulyo freeze-drier (Edwards, UK) operating at 0.01 atm and −60°C. Each batch was prepared in triplicate.

Unmodified LPP without helper excipient (unmodified LPP), blank LPP without dec-ODN (blank LPP_DPPC_ and blank LPP_PEI_) and LPP containing *scramble* dec-ODN (*scramble* LPP_DPPC_ and *scramble* LPP_PEI_) were prepared as controls.

For Confocal Laser Scanning Microscopy (CLSM), labeled LPP (FITC-LPP) were prepared with a fluorescent PLGA (5% w/w) covalently coupled with fluorescein isothiocyanate (FITC) at carboxylic end-group (PLGA-FITC).

Particle shape and external morphology were analysed by Scanning Electron Microscopy (SEM) (Leica S440, Germany) as previously described [22]. The internal microstructure of FITC-labeled LPP_PEI_ was investigated by CLSM analysis carried out on a LSM 510 Zeiss confocal inverted microscope equipped with a Zeiss 63/1.25 oil objective lens (Carl Zeiss, Germany). An argon laser (excitation = 541 nm; emission = 572 nm) was used.

The mean geometric diameter and size distribution of the particles were determined by laser light scattering (Coulter LS 100Q, USA) on a dispersion of freeze-dried microspheres in 0.2% w/v aqueous PVA. Particle size is expressed as volume mean diameter ± S.E.M. of values collected from three different batches.

Powder density was evaluated by a tapped density tester according to European Pharmacopoeia 7 ed. (Ph.Eur.), whereas theoretical mass mean aerodynamic diameter (MMAD_t_) was estimated on the basis of the definition:
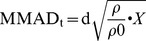
(1)where d is the geometric mean diameter, ρ_0_ is a reference density of 1 g/ml and *X* is the dynamic shape factor, which is 1 for a sphere. In the case of porous particles of approximately spherical shape an approximate bulk measure of ρ is provided by tapped density [22].

### Particle Entrapment Efficiency

The amounts of dec-ODN and PEI encapsulated within LPP_PEI_ were determined after particle degradation in 0.5 N NaOH. For dec-ODN, 10 mg of LPP were dissolved in 2 ml of 0.5 N NaOH under stirring at 37°C until a limpid solution was obtained. Dec-ODN content in the resulting solution was quantified by UV spectrophotometric analysis using a Shimadzu 1204 apparatus (Shimadzu, Milan, Italy) operating at 265 nm. The linearity of the response was verified over the concentration range 0.020–1.0 nmol/ml (r^2^>0.99). Blank LPP were used as control to verify that particle components did not interfere with dec-ODN quantification. The amount of PEI in the solution was contemporary quantified by UV spectrophotometric analysis at 285 nm after complexation with cupper(II) [39]. Results are expressed as encapsulation efficiency (ratio of actual and theoretical loadings × 100) ± S.E.M. of values collected from three different batches.

### *In vitro* Aerosolization Properties

The aerosolization properties of LPP_PEI_ were tested *in vitro* using an Astra Type Multi-Stage Liquid Impinger (MSLI), Type ALI 1000 (Erweka, Italy) after delivery from Turbospin®, a breath-activated, reusable DPI that works with single unit capsule (PH&T Pharma, Milano, Italy). For each test, a hard gelatin capsule (size 2, Capsugel) was filled with about 20 mg of the powder and placed in the Turbospin®. Briefly, 20 ml of methylene chloride were poured into each of the four stages of the impinger to wet the collection surfaces. The capsule was then pierced and the liberated powder drawn through the impactor operating at 60 l/min for 4 s using an electronic digital flow meter (DFM mode). This allowed the aspiration of 4 L of air through the apparatus as recommended by Ph.Eur. The powder deposited on the four MSLI stages, in the induction port and on the final collection site (i.e. filter) was recovered by washing with methylene chloride. In each case, after removal of the solvent by rotary evaporation (Heidolph Instrument, Laborota 4010-digital) the resulting precipitate was dissolved in 1.2 ml of methylene chloride and dec-ODN was extracted into 1.2 ml of water. The suspension was centrifuged (5000 rpm, room temperature, 15 min) and the supernatant analysed for dec-ODN content in the aqueous phase by spectrophotometric analysis as described above.

The emitted dose (ED) was calculated by accurately weighing the capsule before and after Turbospin® actuation. Results are expressed as percentage of powder actually delivered ± S.E.M. of values collected from three different batches.

The fine particle fraction (FPF), that is the fraction of particles with an aerodynamic diameter lower than 5 µm, the experimental mass median aerodynamic diameter (MMAD_exp_) and the geometric standard deviation (GSD) were calculated according to Ph.Eur. deriving a plot of the cumulative mass of powder detained at each stage (expressed as percent of total mass recovered in the impactor) *versus* the cut-off diameter at that stage.

### *In vitro* Release Studies

The *in vitro* release of dec-ODN from LPP was monitored by membrane dialysis in phosphate buffer saline (PBS, 120 mM NaCl, 2.7 mM KCl, 10 mM phosphate salts at pH 7.2 and 37°C). A known amount of particles (8 mg) was suspended in 0.35 ml of PBS and placed in a dialysis membrane bag (MWCO: 50000 Da, Spectra/Por®). The sample was dropped into 4.0 ml of PBS (sink condition), and kept at 37°C. At scheduled time intervals, 1.5 ml of external medium was withdrawn and replaced by the same amount of fresh PBS. The withdrawn medium was analyzed for dec-ODN content by spectrophotometric analysis as described above. Experiments were run in triplicate for each time point of release kinetics.

### Cell Culture

Human epithelial bronchial IB3-1 cells (with ΔF508 CFTR mutation) as well as human epithelial pulmonary NCI-H292 cancer cells (over-expressing MUC2 gene) (American Tissue Culture Catalogue) were purchased by LGC Standards (Sesto San Giovanni, Milan, Italy).

IB3-1 cells were cultured at 37°C in humidified 5%CO_2_/95% air in LHC-8 medium with 5% fetal bovine serum. Petri dishes as well as multiwells were pre-coated with albumin 1 mg/ml, collagen 3 mg/ml and fibronectin 1 mg/ml. The cells were plated in 48 culture wells at a density of 125×10^4^ cells/ml per well or in 10-cm-diameter culture dishes at a density of 5×10^6^ cells/ml per dish and allowed to adhere for 2 h.

NCI-H292 cells were cultured at 37°C in humidified 5% CO2/95% air in RPMI containing 10% fetal bovine serum, 2 mM glutamine, 100 UI/ml penicillin, 100 µg/ml streptomycin, 25 mM Hepes, 1 mM glucose, 0.5 mM sodium bicarbonate and 5 mM sodium pyruvate. The cells were plated in 24 culture wells at a density of 2.5×10^5^ cells/ml/well or 10 cm diameter culture dishes at a density of 3×10^6^ cells/ml/dish and allowed to adhere for 2 h.

After adhesion, the medium was replaced with fresh medium and cells (IB3-1 or NCI-H292 cells) were stimulated with LPS from *P. aeruginosa* (10 µg/ml) for 24 and 72 h in the absence or presence of naked dec-ODN (2.0 µM), LPP_PEI_ containing scramble dec-ODN (2.0 µM), LPP_PEI_ containing dec-ODN (2.0 µM) or the same amount of blank LPP_PEI_.

### Cytotoxicity in NCI-H292 Cells

The cytotoxicity of LPP_PEI_ was determined by using 3-(4,5-dimethylthiazol-2yl)-2,5-diphenyl-2H-tetrazolium bromide (MTT) conversion assay. Briefly, NCI-H292 cells were incubated as described above. Thereafter the medium was replaced with fresh medium and cells were stimulated with LPS from *P. aeruginosa* (10 µg/ml) for 24 h in the presence of either naked dec-ODN (2.0 µM), PEI alone (0.1 mg, corresponding to the amount of PEI present in 2.0 µM LPP_PEI_), dec-ODN (2.0 µM)/PEI (0.1 mg), LPP prepared without PEI (dec-ODN 2.0 µM; 10 mg/ml), LPP_PEI_ (dec-ODN 2.0 µM; 10 mg/ml) or blank LPP_PEI_ (10 mg/ml). The optical density (OD) of each well was measured with a microplate spectrophotometer (Titertek Multiskan MCCC/340) equipped with a 620 nm filter. The cell viability in response to the treatment with test compounds was calculated as % dead cells = 100 − (OD treated/OD control) × 100.

### Stability of Dec-ODN Released from LPP_PEI_

The stability of dec-ODN was evaluated by gel electrophoresis on naked dec-ODN or LPP_PEI_ containing dec-ODN (2.0 µM) in NCI-H292 cells. At 24, 48 and 72 h, cell culture medium was collected and centrifuged (14000 rpm, 4°C, 20 min). dec-ODN was extracted from the pellet by a solvent extraction method employing methylene chloride and water [21]. Then, the supernatants containing either released dec-ODN or naked dec-ODN as well as dec-ODN extracted from LPP_PEI_ were loaded into 1% agarose gel in TBE buffer (100V, 15 min). Freshly annealed naked dec-ODN was used as internal control. In each case, dec-ODN was visualized by ethidium bromide staining. Images were captured by ImageQuant 400 (GE Healthcare).

### Cytosolic and Nuclear Extracts

Cytosolic and nuclear extracts of NCI-H292 cells were prepared as previously described with some modifications [21]. Briefly, harvested cells (3×10^6^) were washed two times with ice-cold PBS and centrifuged at 180 *g* for 10 min at 4°C. The cell pellet was resuspended in 100 µl of ice-cold hypotonic lysis buffer (10 mM Hepes, 10 mM KCl, 0.5 mM phenylmethylsulphonyfluoride, 1.5 µg/ml soybean trypsin inhibitor, 7 µg/ml pepstatin A, 5 µg/ml leupeptin, 0.1 mM benzamidine, 0.5 mM dithiothreitol) and incubated on ice for 15 min. The cells were lysed by rapid passage through a syringe needle for 5 or 6 times and the cytoplasmic fraction was then obtained by centrifugation for 1 min at 13,000 *g*. The supernatant containing the cytosolic fraction was removed and stored at –80°C. The nuclear pellet was resuspended in 60 µl of high salt extraction buffer (20 mM Hepes pH 7.9, 10 mM NaCl, 0.2 mM EDTA, 25% v/v glycerol, 0.5 mM phenylmethylsulphonyfluoride, 1.5 µg/ml soybean trypsin inhibitor, 7 µg/ml pepstatin A, 5 µg/ml leupeptin, 0.1 mM benzamidine, 0.5 mM dithiothreitol) and incubated with shaking at 4°C for 30 min. The nuclear extract was then centrifuged for 15 min at 13,000 *g* and supernatant was aliquoted and stored at −80°C. Protein concentration was determined by Bio-Rad (Milan, Italy) protein assay kit.

### Electrophoretic Mobility Shift Assay (EMSA)

Double stranded ONs containing the NF-κB recognition sequences (5′-CAACGGCAGGGGAATCTCCCTCTCCTT-3′) were end-labeled with 32P-γ-ATP. Nuclear extracts containing 5 µg protein were incubated for 15 min with radiolabeled ONs (2.5–5.0×104 cpm) in 20 µl reaction buffer containing 2 µg poly dI-dC, 10 mM Tris-Hcl (pH 7.5), 100 mM NaCl, 1 mM EDTA, 1 mM dithiothreitol, 10% (v/v) glycerol. The specificity of the DNA/protein binding was determined by competition reaction in which a 50-fold molar excess of unlabeled wild-type, mutant or Sp-1 ON was added to the binding reaction 15 min before radiolabeled probe. In supershift assay, antibodies reactive to p50 or p65 or a mixture of both were added to the reaction 15 min before the addition of radiolabeled probe. Nuclear protein-ON complexes were resolved by electrophoresis on a 6% non-denaturing polyacrylamide gel in 1× TBE buffer at 150V for 2 h at 4°C. The gel was dried and autoradiographed with intensifying screen at −80°C for 20 h. Subsequently, the relative bands were quantified by densitometric scanning of the X-ray films with a GS-700 Imaging Densitometer (Bio-Rad) and a computer program (Molecular Analyst, IBM).

### Western Blot Analysis

Cytosolic and nuclear fraction proteins were mixed with gel loading buffer (50 mM Tris, 10% SDS, 10% glycerol, 10% 2-mercaptoethanol, 2 mg/ml of bromophenol) in a ratio of 1∶1, boiled for 3 min and centrifuged at 10,000 *g* for 5 min. Protein concentration was determined and equivalent amounts (30 µg) of each sample were electrophoresed in a 6–12% discontinuous polyacrylamide minigel. The proteins were transferred onto nitro-cellulose membranes, according to the manufacturer’s instructions (Protran, Schleicher & Schuell, Dassel, Germany). The membranes were saturated by incubation at room temperature for 2 h with 10% non-fat dry milk in PBS and then incubated with (1∶1000) anti-MUC2, anti-p50 or anti-p65 at 4°C overnight. The membranes were washed three times with 0.1% Tween 20 in PBS and then incubated with anti-rabbit or anti-mouse immunoglobulins coupled to peroxidase (1∶1000) (DAKO, Milan, Italy). The immunocomplexes were visualised by the ECL chemiluminescence method (Amersham, Milan, Italy). The membranes were stripped and re-probed with β-actin (for cytosolic protein extracts) or GAPDH (for nuclear protein extracts) antibodies to verify equal loading of proteins. Subsequently, the relative expression of MUC2, p50 or p65 proteins in cytosolic and nuclear fraction was quantified by densitometric scanning of the X-ray films with a GS 700 Imaging Densitometer and a computer programme (Molecular Analyst, IBM).

### Reverse Transcription-polymerase Chain Reaction

Total RNA extraction from NCI-H292 cells, by using TRIzol, was performed as described elsewhere [21]. MUC2 mRNA levels were determined by using the reverse transcriptase polymerase chain reaction (RT-PCR) method. The housekeeping gene GAPDH was used as an internal control. Five micrograms of total RNA were reverse-transcribed into cDNA by using oligo (dT)12–18 primer (Invitrogen) and MMLV-Reverse Transcriptase (Invitrogen). One microliter of cDNA was amplified by PCR using Taq Polymerase (Invitrogen) under the following conditions: a first cycle at 94°C for 1 min 40 s, 25 cycles at 94°C for 40 s, annealing at 56°C for 40 s, extension at 72°C for 1 min and one additional cycle of extension at 72°C for 8 min. The primers were: for MUC-2, 5′-CCA TGC GTG CCT CTC TGC AA (forward) and 5′-GTG GGT TGG GTG ACA CAC TC-3′ reverse; for IL-8, 5′-TGC CAA GGA GTG CTA AAG-3′ (forward) e 5′-TCT CAG CCC TCT TCA AAA-3′ (reverse); for GAPDH, 5′-CGC TGA GTA CGT GGA G-3′ (forward) and 5′-GAG GAG TGG GTC TCG CTG TT-3′(reverse). The PCR products were run on a 1% agarose gel and visualised by Sybr Safe staining.

### Statistics

Results were expressed as the mean ± S.E.M. of n experiments. Statistical significance was calculated by one way analysis of variance (ANOVA) and Bonferroni-corrected p value for multiple comparison test. The level of statistically significant difference was defined as p<0.05.

## Supporting Information

Figure S1 Representative ELISA shows IL-6 and IL-8 protein levels induced by LPS in IB3-1 cells at 24 and 72 h. Data are expressed as mean ± S.E.M. of three experiments. °°°p<0.001 *vs*. unstimulated cells; **p<0.01; ***p<0.001 *vs*. LPS.
(TIF)

Figure S2 Agarose gel electrophoresis of LPP_DPPC_ or naked dec-ODN collected from cell culture medium at 24, 48 and 72 h. (A) dec-ODN released from LPP_DPPC_ or naked dec-ODN from supernatant; (B) dec-ODN extracted from LPP_DPPC_ pellet and annealed naked dec-ODN (internal control). Data are from a single experiment and are representative of three separate experiments.
(TIF)

Supporting Information S1 Effects of LPP_DPPC_ containing dec-ODN on IL-6/IL-8 secretion from CF cells - Integrity of dec-ODN in cultured CF cells.
(DOC)
