# Supplementary figures and images for: PEI-Engineered Respirable Particles Delivering a Decoy Oligonucleotide to NF-κB: Inhibiting MUC2 Expression in LPS-Stimulated Airway Epithelial Cells
Source: PLoS One. 2012 Oct 3;7(10):e46457. doi: 10.1371/journal.pone.0046457 (PMC3463602; doi:10.1371/journal.pone.0046457)

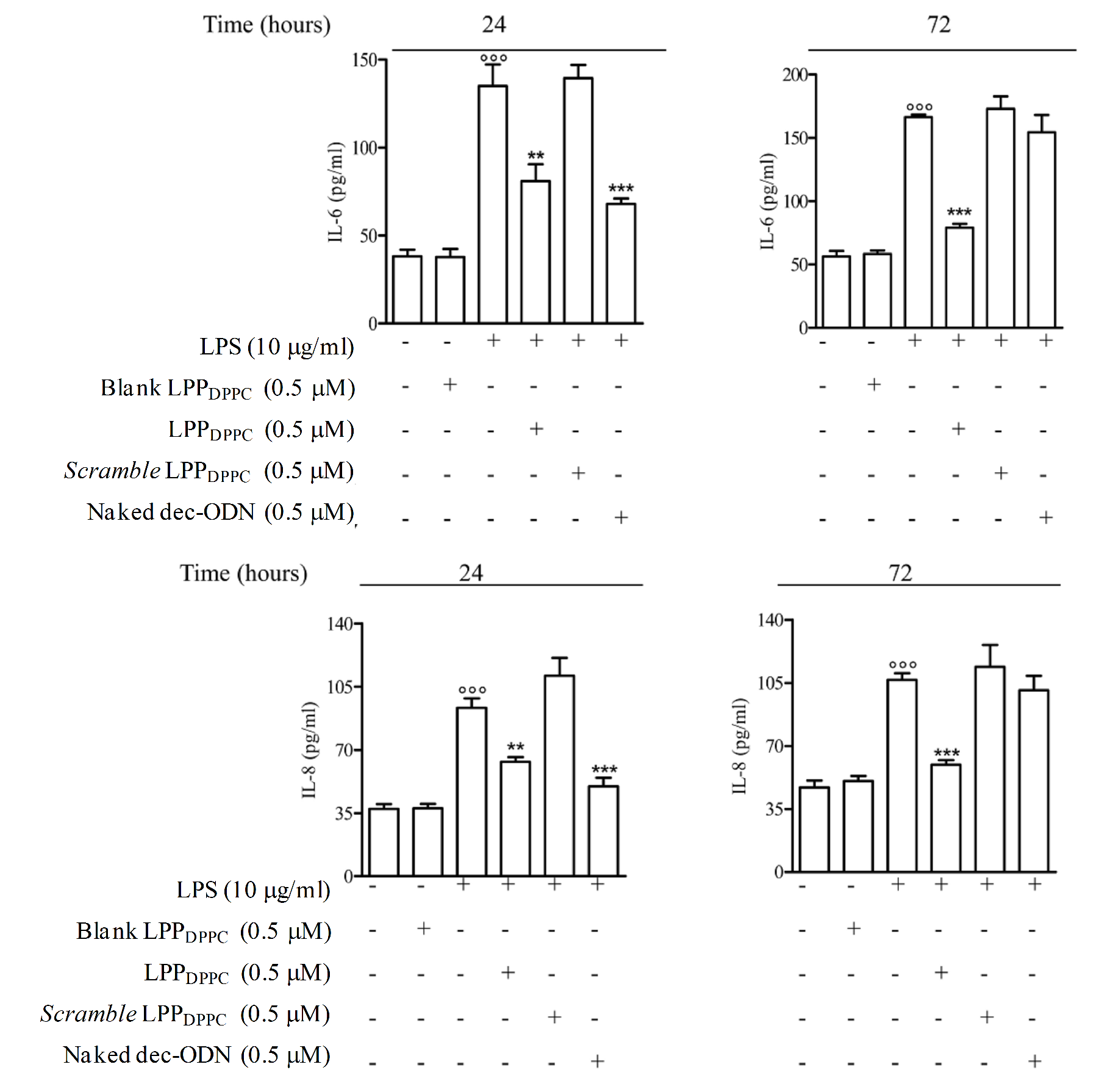

Supplement: Figure S1 — Representative ELISA shows IL-6 and IL-8 protein levels induced by LPS in IB3-1 cells at 24 and 72 h. Data are expressed as mean ± S.E.M. of three experiments. °°°p<0.001 vs. unstimulated cells; **p<0.01; ***p<0.001 vs. LPS. (TIF) [file pone.0046457.s001.tif]

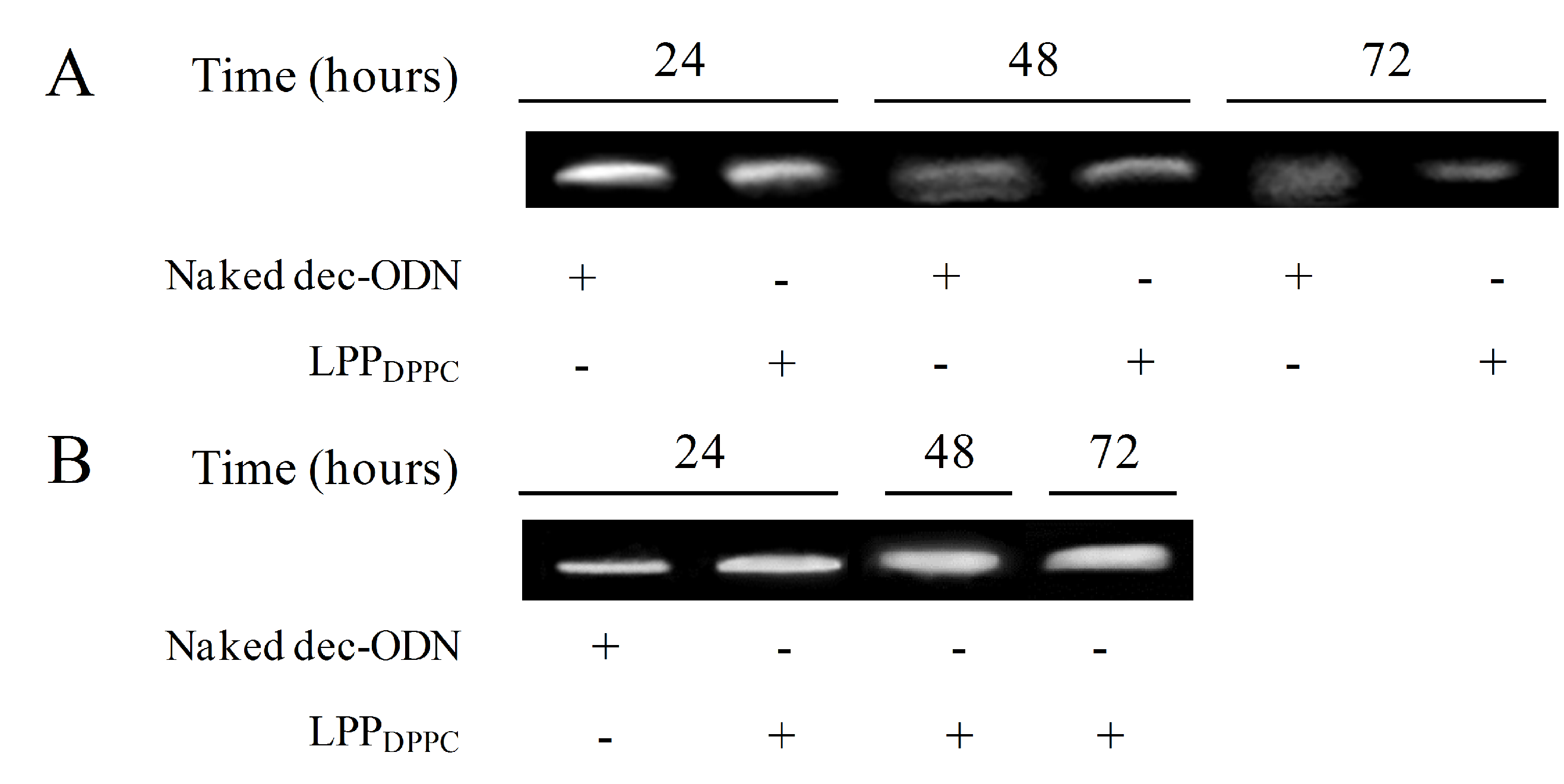

Supplement: Figure S2 — Agarose gel electrophoresis of LPPDPPC or naked dec-ODN collected from cell culture medium at 24, 48 and 72 h. (A) dec-ODN released from LPPDPPC or naked dec-ODN from supernatant; (B) dec-ODN extracted from LPPDPPC pellet and annealed naked dec-ODN (internal control). Data are from a single experiment and are representative of three separate experiments. (TIF) [file pone.0046457.s002.tif]
