# Supplementary material for: PEI-Engineered Respirable Particles Delivering a Decoy Oligonucleotide to NF-κB: Inhibiting MUC2 Expression in LPS-Stimulated Airway Epithelial Cells
Source: PLoS One. 2012 Oct 3;7(10):e46457. doi: 10.1371/journal.pone.0046457 (PMC3463602; doi:10.1371/journal.pone.0046457)
Supplement: Supporting Information S1 — Effects of LPPDPPC containing dec-ODN on IL-6/IL-8 secretion from CF cells - Integrity of dec-ODN in cultured CF cells. (DOC) [file pone.0046457.s003.doc]

# Polyethylenimine-engineered respirable particles delivering a decoy oligonucleotide to NF-κb: a strategy to inhibit MUC2 expression in LPS-stimulated human airway epithelial cells

Francesca Ungaro^1*§^, Daniela De Stefano^2*^, Concetta Giovino^1^, Alessia Masuccio^2^, Agnese Miro^1^, Raffaella Sorrentino^2^, Rosa Carnuccio^2^, Fabiana Quaglia^1^

^1^*Department of Pharmaceutical and Toxicological Chemistry, University of Naples Federico II, Naples, Italy;* ^2^*Department of Experimental Pharmacology, University of Naples Federico II, Naples, Italy.*

^*^ The authors equally contributed to this work

^§^Corresponding author:

Francesca Ungaro

[ungaro@unina.it](mailto:ungaro@unina.it)

tel. +39 81678667

fax +39 81678630

# SUPPORTING INFORMATION S1

Effects of LPP_DPPC_ containing dec-ODN on IL-6/IL-8 secretion from CF cells

Human epithelial bronchial IB3-1 (with ΔF508 CFTR mutation) cells were cultured at 37°C in humidified 5%CO_2_/95% air in LHC-8 medium with 5% foetal bovine serum. Petri dishes as well as multiwells were pre-coated with albumin 1 mg/ml, collagen 3 mg/ml and fibronectin 1 mg/ml. The cells were plated in 48 culture wells at a density of 125×10^4^ cells/ml per well or in 10-cm-diameter culture dishes at a density of 5×10^6^ cells/ml per dish and allowed to adhere for 2 h. Thereafter the medium was replaced with fresh medium, and cells were stimulated with LPS (10 μg/ml) from *P. aeruginosa* for 24 and 72 h in the absence or presence of *naked* dec-ODN (0.5 μM), LPP_DPPC_ containing *scramble* dec-ODN (scramble LPP_DPPC_) (0.5 μM), LPP_DPPC_ (0.5 μM) and blank LPP_DPPC_ (0.5 μM). The cell viability was determined by using 3-(4,5-dimethylthiazol-2yl)-2,5-diphenyl-2H-tetrazolium bromide (MTT) conversion assay as previously described (De Stefano et al, 2011).

Enzyme-linked immunosorbent assay (ELISA) was performed on supernatants from cells to quantify IL-6 and IL-8 according to the manufacturer’s instructions (SABiosciences, USA). Representative ELISA showing IL-6 and IL-8 protein levels induced by LPS in IB3-1 cells at 24 and 72 h are reported in figure S1. Results are expressed as the means ± S.E.M. of *n* experiments. Statistical significance was calculated by one-way analysis of variance (ANOVA) and Bonferroni-corrected P-value for multiple comparison test. The level of statistically significant difference was defined as p<0.05.

LPS challenge of IB3-1 cells for 24 and 72 h induced a significant increase in the IL-6 and IL-8 protein levels, as compared to unstimulated cells. Treatment of cells with LPP_DPPC_ (0.5 μM) significantly reduced IL-6 and IL-8 protein levels (by 47.17±9.42% and 31.30±5.38%, at 24 h, respectively; by 55.43±6.13% and 56.77±4.59%, at 72 h, respectively; *n=*3). At the same concentration, naked dec-ODN inhibited IL-6 and IL-8 secretion only at 24 h (by 59.70±9.46% and 50.20±4.61%, respectively; *n=*3). *Scramble* LPP_DPPC_ as well as blank LPP_DPPC_ did not exhibit any effect. Cell viability was not affected by any treatment (≥85%).

Integrity of dec-ODN in cultured CF cells

The stability of dec-ODN within LPP_DPPC_ (0.5 µM) or naked dec-ODN (0.5 µM) was evaluated by gel electrophoresis in IB3-1 cell cultures. At 24, 48 and 72 h, cell culture medium was collected and centrifuged (14000 rpm, 4°C, 20 min). dec-ODN was extracted from the pellet by the solvent extraction method described above to evaluate the actual loading of dec-ODN. Then, the supernatants containing either released dec-ODN or naked dec-ODN as well as dec-ODN extracted from LPP were loaded into 1% agarose gel in TBE buffer (100V, 15 min). Freshly annealed naked dec-ODN was used as internal control. In each case, dec-ODN was visualized by ethidium bromide staining. Imageswere captured by ImageQuant 400 (GE Healthcare).

As shown in figure S2, dec-ODN released from LPP_DPPC_ at 24 h exhibited a marked band, the intensity of which was reduced at 48 and 72 h. In contrast, the band of naked dec-ODN was evident at 24 h and smeared at 48 and 72 h, suggesting partial degradation. Furthermore, the band of dec-ODN extracted from LPP_DPPC_ pellet (Figure S2, panel B) showed a long-lasting protection of dec-ODN by LPP_DPPC_ at all time points. Annealed naked dec-ODN is reported as an internal control.

**Reference**

De Stefano D, Ungaro F, Giovino C, Polimeno A, Quaglia F, Carnuccio R (2011) Sustained inhibition of IL-6 and IL-8 expression by ODN decoy to NF-kappaB delivered through respirable large porous particles in LPS-stimulated cystic fibrosis bronchial cells. J Gene Med 13: 200-208.
